# Mosquitoes in the Danube Delta: searching for vectors of filarioid helminths and avian malaria

**DOI:** 10.1186/s13071-017-2264-8

**Published:** 2017-07-05

**Authors:** Angela Monica Ionică, Carina Zittra, Victoria Wimmer, Natascha Leitner, Jan Votýpka, David Modrý, Andrei Daniel Mihalca, Hans-Peter Fuehrer

**Affiliations:** 1 Department of Parasitology and Parasitic Diseases, University of Agricultural Sciences and Veterinary Medicine Cluj-Napoca, 335700 Cluj-Napoca, Romania; 2 Department of Pathobiology, Institute of Parasitology, University of Veterinary Medicine Vienna, Vienna, Austria; 3 Department of Parasitology, Faculty of Science, Charles University, Prague, Czech Republic; 4 Department of Pathology and Parasitology, University of Veterinary and Pharmaceutical Sciences, Brno, Czech Republic; 5 CEITEC-VFU, University of Veterinary and Pharmaceutical Sciences, Brno, Czech Republic; 6 Institute of Parasitology, Biology Centre of Czech Academy of Sciences, České Budĕjovice, Czech Republic

**Keywords:** Danube Delta, Filarioids, Avian malaria, Mosquito vectors

## Abstract

**Background:**

Mosquitoes are arthropods of major importance to animal and human health because they are able to transmit pathogenic agents such as filarioids (Spirurida), vector-borne nematodes, which reside in the tissues of vertebrates. In Europe, recent research has mostly focused on mosquito-borne zoonotic species, while others remain neglected. Mosquitoes are also vectors of avian malaria, which has an almost worldwide distribution, and is caused by several *Plasmodium* species and lineages, the most common being *P. relictum*. The Danube Delta region of Romania is one of the most important stopover sites for migratory birds. The local mosquito fauna is diverse and well represented, while filarial infections are known to be endemic in domestic dogs in this area. The aim of the present study was thus to assess the potential vector capacity for various filarial helminths and avian malaria of mosquitoes trapped in the Danube Delta.

**Methods:**

In July 2015, mosquitoes were collected at seven sites located in and around a rural locality in the Danube Delta region of Romania, using CO_2_-baited traps and hand aspirators. Additionally, a trap was placed next to a microfilaremic dog co-infected with *Dirofilaria repens* and *D. immitis*. All randomly trapped mosquitoes were identified to the species level and pooled according to date, sampling site, and taxon. Three hundred individual mosquitoes sampled next to the microfilaremic dog were processed individually and divided into abdomen and thorax/head. Following DNA extraction, all samples were screened for the presence of DNA of filarioid helminths and avian malaria agents by PCR techniques.

**Results:**

All 284 pools (a total of 5855 mosquitoes) were negative for filarioid DNA. One pool of *Culex modestus* mosquitoes was positive for *Plasmodium* sp. lineage Donana03. In the individually extracted mosquitoes, one abdomen of *Aedes vexans* was positive for *D. repens* DNA, one thorax/head of *Ae. vexans* was positive for DNA of *Setaria labiatopapillosa*, and two thorax/head of *Cx. pipiens* f. *pipiens* were positive for *P. relictum* lineage pSGS1.

**Conclusion:**

The present study suggests the vector competence of *Cx. modestus* and *Cx. pipiens* for avian *Plasmodium* including pathogenic species *P. relictum* and *Ae. vexans* for mammalian filarioids. Moreover, it indicates the role of *Cx. pipiens* f. *pipiens* as a potential natural vector of *P. relictum* lineage pSGS1 in nature.

## Background

Mosquitoes (Diptera: Culicidae) are a diverse group of bloodsucking arthropods comprising 44 genera including approximately 3500 species and subspecies [1]. Overall, they are considered a key threat to animal and human health [2]. The genera *Aedes*, *Culex* and *Anopheles* show a high diversity of species, including the vectors of pathogens causing infections relevant for public health, such as Rift Valley fever, dengue fever, yellow fever, Zika, chikungunya, malaria and filarioses [3].

The Danube Delta is situated in eastern Romania and comprises thirty types of ecosystems [4]. This area is also characterized by a significant biodiversity, comprising over 1800 plant and 3500 animal species [4]. The specific landscape (marshes, lakes and channels), along with the local climatic conditions provide optimal conditions for the development of an abundant and well represented mosquito fauna, comprising 31 species from 10 genera [4].

Filarioids (Spirurida) are parasitic vector-borne nematodes, residing in the tissues of all classes of vertebrates, except fish [5]. Several species which act as agents of human disease have been extensively studied [6], while others are still neglected, due to their minimal clinical importance. Among mosquito-borne filarioids in Europe, increased attention has been given to zoonotic species, parasites of canids, namely *Dirofilaria immitis*, which causes a severe and life-threatening cardiopulmonary disease of domestic dogs [7], and *D. repens*, which resides in the subcutaneous tissues, often asymptomatic and sometimes associated with a variety of dermatological conditions [8]. There are several species of mosquitoes, mainly in the genera *Aedes*, *Culex* and *Anopheles*, that allow larval development of both *Dirofilaria* species and were found to harbour infective larvae, thus having a confirmed vectorial capacity, as reviewed by Simón et al. [7]. Species of the genus *Setaria* are mosquito-borne filarioids which occur in the abdominal cavity of artiodactyls (particularly Bovidae), hyracoids and equines [5]. Except for *S. tundra*, the adults are generally non-pathogenic or associated with fibrinous peritonitis, while larvae may migrate erratically and produce neuropathological disorders in unusual hosts [9–11]. In Romania, five species of filarioids parasitizing domestic or wild carnivores have been identified so far: *D. immitis*, *D. repens*, *Acanthocheilonema reconditum* [12, 13], *Onchocerca lupi* [14] and *Cercopithifilaria bainae* [15]. Except for *O. lupi*, all the other species are known to be endemic in the Danube Delta Region [12, 13, 15]. However, with the exception of a few case reports published in the first half of the twentieth Century [16–20], there is no national data regarding the occurrence of other species of filarial parasites (e.g. *Setaria* spp., *Parafilaria* spp., *Onchocerca* spp., etc.) in non-carnivorous mammals.

Avian *Plasmodium* species are the most prevalent and widespread vertebrate malarial agents, with an almost worldwide distribution [21]. One of the most studied avian malaria parasite is *Plasmodium relictum*, which is the most prevalent parasite among avian plasmodia, having numerous genetic lineages [22]. Under laboratory conditions, complete sporogony of this parasite was described in *Cx. pipiens* f. *molestus* (lineages pGRW4, pSGS1 and pGRW11) and *Cx. quinquefasciatus* (pSGS1, pGRW4) [22–24]. Members of the *Cx. pipiens* complex are suggested to be the most important vectors for *P. relictum* in the field. Moreover, several other avian *Plasmodium* species and lineages are known to be present in Europe (e.g. [25, 26]). The Danube Delta is one of the most important stopover sites for migratory birds, with an estimated two million individuals belonging to three hundred avian species using the region’s ecosystems each year [27]. However, no records of *Plasmodium* infection in birds have been provided for this region so far.

The aim of the present study was to assess the potential vector capacity of mosquitoes trapped in the Danube Delta, for filarial helminths and avian malaria.

## Methods

### Study area and sampling

During the EurNegVec Training School “Vector-Borne Diseases and One Health”, mosquitoes were collected (between 10 and 14 July 2015) in the Danube Delta region of Romania, at seven locations in and around a rural locality, Chilia Veche (45.421944N, 29.289722E). Mosquitoes were collected with carbon dioxide baited BG Sentinel™ traps (Biogents AG, Regensburg, Germany), CO_2_-baited CDC traps (model 512 and 1012, John W. Hock Company, Gainesville, FL, USA) and by hand aspirators. Additionally, a carbon dioxide baited BG Sentinel™ trap (Biogents AG, Germany) was placed next to a microfilaremic dog known to be co-infected with *D. immitis* and *D. repens*. All captured mosquitoes were stored at -20 °C and brought to the Institute of Parasitology of the University of Veterinary Medicine Vienna.

### Mosquito identification and processing

All mosquitoes were identified to species level using morphological keys available in the literature [2].

After identification, all randomly trapped mosquitoes (*n* = 5855) were grouped and pooled according to species, capture date and sampling site. When necessary, some groups were further subdivided, so that each final pool would contain a maximum of 25 individual mosquitoes. To each pool, 2–3 Precellys Ceramic Beads 2.8 mm (Peqlab, Erlangen, Germany) were added before homogenization, using a TissueLyserII (Qiagen, Hilden, Germany). DNA was extracted from homogenates, using the innuPREP DNA Mini Kit (Analytik Jena, Jena, Germany) according to the manufacturer’s instructions.

In contrast, 300 mosquitoes from the trap placed next to the microfilaremic dog were processed individually. The abdomen was separated from the thorax/head of each identified mosquito, and both parts were separately homogenized as mentioned above. DNA was extracted using the ZR-Duet™ DNA/RNA MiniPrep Kit (Zymo Research Corp.,Irvine, CA, USA) according to the manufacturer’s instructions.

The screening for filarioid helminths was performed in both the pools and the individually processed mosquitoes by PCR amplification of a 724 bp fragment of the mitochondrial cytochrome *c* oxidase subunit 1 gene (flanking the entire barcode region), using the generalistic H14FilaCOIFw/H14FilaCOIRv primer pair, as previously described [28]. The presence of DNA of avian *Plasmodium* and *Haemoproteus* was assessed by means of nested PCR, targeting a 667 bp fragment of the mitochondrial cytochrome *b* gene, according to a previously described protocol [29].

All PCR-positive samples were sequenced using an external service (LGC Genomics GmbH, Germany). The attained sequences were compared to those available in the GenBank database using Basic Local Alignment Tool (BLAST) analysis. Furthermore, avian *Plasmodium* sequences were compared to others available in the MalAvi database [30].

In the case of positivity for pathogens, mosquitoes belonging to the *Cx. pipiens* complex were further analysed molecularly in order to differentiate *Cx. pipiens* from *Cx. torrentium* (*ace-2* gene) and *Cx. pipiens* f. *pipiens* from *Cx. pipiens* f. *molestus* (CQ11 locus), as previously reported [31–33].

## Results

### Mosquito pools

Overall, 284 pools comprising 5855 mosquitoes belonging to 11 species included in six genera (Table 1) which are known to be present in the Danube Delta region [4, 34], were screened for the presence of DNA of filarioid helminths and avian malaria. All samples were negative for filarioids. One pool of *Cx. modestus* mosquitoes collected on 11th of July (45.413038N, 29.279688E) was positive for *Plasmodium* DNA. Sequence analysis revealed a 100% homology with a *Plasmodium* sp. of the lineage Donana03 (GenBank: JX458328), which has so far not been assigned to a particular morphospecies [25]. The sequence was deposited in the GenBank database under the accession number KX570600.

**Table 1:** Number and relative abundance of trapped mosquitoes collected in the Danube Delta, including their division in pools

Species	*n*	%	Pools
*Aedes vexans*	2774	47.38	116
*Coquillettidia richiardii*	1725	29.46	74
*Culex pipiens* (*sensu* *lato*)	752	12.84	34
*Aedes* sp.	253	4.32	13
*Ochlerotatus caspius*	108	1.84	10
*Anopheles* sp.	79	1.35	9
*Culex martinii*	44	0.75	6
*Anopheles maculipennis*	37	0.63	7
*Culex* sp.	32	0.55	3
*Anopheles plumbeus*	29	0.50	4
*Culex modestus*	14	0.24	3
*Anopheles claviger*	5	0.09	2
*Anopheles hyrcanus*	2	0.03	2
*Culiseta annulata*	1	0.02	1
Total	5855	100	284

### Individual mosquitoes

From the 300 mosquitoes that were trapped next to the microfilaremic dog (Table 2), one abdomen and one thorax/head from two different individuals of *Ae. vexans* were positive for filarial DNA. Sequence analyses of the abdomen sample revealed the presence of *D. repens* (100% similarity to several other European isolates, e.g. KR998257), while the thorax/head sample was positive for *Setaria labiatopapillosa* (99% similarity to AJ544872). Two thorax/head samples from mosquitoes belonging to the *Cx. pipiens* complex were positive for DNA of *P. relictum* pSGS1 (100% similarity to several other isolates, e.g. KU752590). All four sequences were deposited in GenBank (accession numbers KX570597–KX570599 and KX570601).

**Table 2:** Mosquitoes collected next to a microfilaremic dog positive for *D. repens* and *D. immitis*

Species	*n*	%
*Coquillettidia richiardii*	139	46.34
*Aedes vexans*	75	25.00
*Culex pipiens* (*sensu lato*)	67	22.33
*Ochlerotatus caspius*	9	3.00
*Anopheles* sp.	8	2.67
*Anopheles plumbeus*	1	0.33
*Anopheles hyrcanus*	1	0.33
Total	300	100

Both positive specimens belonging to the *Cx. pipiens* complex were molecularly identified as *Cx. pipiens* f. *pipiens*.

## Discussion

Mosquito-borne filarioids (e.g. *Dirofilaria* spp., *Setaria* spp.) can be transmitted by several species of mosquitoes, including the *Cx. pipiens* complex, *Coquillettidia richiardii* and *Ae. vexans* [10, 35, 36], which were trapped and examined in large numbers during the present study. Overall, due to the advantages it presents, xenomonitoring of mosquitoes for filarioid species has been largely used throughout Europe during the past decade [37–44]. However, the attained results may depend on the employed PCR techniques, without necessarily reflecting the actual epidemiological situation [44]. Surprisingly, in the present study, all mosquito pools were negative for filarial DNA, despite the high prevalence of *Dirofilaria* spp. infection in dogs originating from the same area [12]. In neighboring countries, the overall positivity rate of mosquito pools for various species of filarioids was above 30% [42, 43]. In Hungary, an overall prevalence of filarioid DNA was of 36.8% in the tested mosquito pools, with *D. repens* DNA having been detected in eight mosquito species, *S. tundra* DNA in four mosquito species and the DNA of an unidentified filarioid was found in one pool of *Cx. pipiens* [42]. In the Republic of Moldova, 26.51% of tested pools were positive for *D. repens* DNA, which was identified in 17 mosquito species, while DNA of *D. immitis* was identified only in four mosquito species, with a prevalence of 8.64%, suggesting a broad spectrum of local potential vector species [43]. However, these studies [42, 43] present the outcome of longitudinal multiannual monitoring, while the present one includes a single sampling event, a case in which negativity may be due to the fact that the mosquitoes were caught within a short time span, that they had never fed previously, or fed on other hosts than dogs.

From the individual samples, one abdomen of *Ae. vexans* was positive for *D. repens* DNA. However, this indicates an infected, but not an infective individual, pointing out the fact that the mosquito had recently fed on a positive dog [45]. Theoretically, this mosquito species could be a competent vector for *D. repens*, but several studies suggested it is not particularly attracted to dogs as hosts, thereby playing a minor role in the epidemiology of this parasite [46–48].

A thorax/head sample of *Ae. vexans* was positive for DNA of *S. labiatopapillosa*. This species of filarioid normally resides in the peritoneal cavity of bovines, with no apparent associated pathology [10], which may account for the general scarcity of available epidemiological data. However, when infecting unusual hosts (e.g. sheep, horses), it may become pathogenic and is associated with lesions in the central nervous system, leading to unspecific neurological conditions [10]. To the best of our knowledge, so far, the only confirmed cases of human infection were recorded in Bucharest (south-eastern Romania), where four patients living in the same area presented with ocular infections [49]. *Aedes vexans* seems to be one of the most efficient natural vectors for this species of filarioid [10]. The microfilariae develop and become infective in the flight muscles [50, 51]. As the actual localization of the larvae in our sample (thorax-developing or head/proboscis-infective) is unknown, our result solely indicates vector competence, but does not further confirm it. However, considering the zoonotic nature of this parasite, further investigations regarding its occurrence in definitive hosts and potential vectors should be undertaken.

Interestingly, the only parasite identified in the pooled samples was *Plasmodium* sp. lineage Donana03 in *Cx. modestus*. This finding confirms a previous study, where the same lineage was documented in the same mosquito species in southern Spain [25]. However, in this case, as the sampling was performed in pools, vector competence is not proven, but suggested. In contrast, *P. relictum* lineage pSGS1 was identified in the head/thorax, but not in the abdomen, of two *Cx. pipiens* f. *pipiens* mosquitoes. The presence of *P. relictum* lineage pSGS1 DNA has been demonstrated in field-collected *Cx. pipiens* mosquitoes from various European countries (e.g. [52–54]), but in those studies the form was not identified. As mentioned above, the mammalophilic form *Cx. pipiens* f. *molestus* is a proven vector of this lineage under laboratory conditions [24]. However, until now, the ornithophilic form, *Cx. pipiens* f. *pipiens* cannot be bred under laboratory conditions, and therefore our results indicate that this form might be an important vector of *P. relictum* pSGS1 in the field.

## Conclusion

The present study suggests vector competence of *Cx. modestus* for avian *Plasmodium* spp., of *Ae. vexans* for mammalian filarioids in the Danube Delta and indicates the role of *Cx. pipiens* f. *pipiens* as potential vector of *P. relictum* lineage pSGS1 in nature.
